# Altered Brain Glucose Consumption in Cogan's Syndrome

**DOI:** 10.1155/2016/3207150

**Published:** 2016-12-06

**Authors:** Paolo Mora, Livia Ruffini, Caterina Ghetti, Stella Ghirardini, Maura Scarlattei, Giorgio Baldari, Carla Cidda, Pierangela Rubino, Stefano A. Gandolfi, Jelka G. Orsoni

**Affiliations:** ^1^Institute of Ophthalmology, University Hospital of Parma, Parma, Italy; ^2^Nuclear Medicine Unit, University Hospital of Parma, Parma, Italy; ^3^Medical Physic Department, University Hospital of Parma, Parma, Italy

## Abstract

*Purpose*. Prospective, controlled cohort study to investigate possible alterations in brain glucose metabolism (CMRglc) in patients with Cogan's syndrome (CS).* Patients and Methods*. Functional mapping of the CMRglc was obtained by quantitative molecular imaging positron emission tomography, combined with computed tomography (FDG-PET/CT). The patients were divided into three clinical groups: typical CS; atypical CS (ACS); autoimmune inner ear disease (AIED). The unmatched control group (CG) consisted of subjects requiring FDG-PET/CT for an extracranial pathology. Statistical mapping searched areas of significant glucose hypometabolism in all the affected patients (DG) and in each clinical subgroup. The results were compared with those of the CG.* Results*. 44 patients were enrolled (DG) and assigned to the three study groups: 8 patients to the CS group; 21 patients to the ACS group; and 15 to the AIED group. Sixteen subjects formed the CG group. Areas of significant brain glucose hypometabolism were identified in all the study groups, with the largest number and extension in the DG and CS.* Conclusions*. This study revealed areas of significantly altered CMRglc in patients with CS (any subform) without neurologic complains and normal conventional neuroimaging. Our results suggest that FDG-PET/CT may represent a very useful tool for the global assessment of patients with Cogan's syndrome.

## 1. Introduction

Cogan's syndrome (CS) is named after the ophthalmologist Cogan, who was the first to report “non-syphilitic interstitial keratitis and audiovestibular symptoms” [1]. The disease was probably present long before this formal identification, with Ludwig von Beethoven perhaps its most prominent sufferer [2]. CS is a rare autoimmune disease characterized by ocular inflammation and sensorineural hearing loss, with a number of possible related findings and a pathogenetic mechanism of vasculitis. Possible combined manifestations are mostly neurological, rheumatological, cardiovascular, and gastrointestinal; the latter two may be very severe, due to aortic insufficiency or mesenteric arteritis [3–8]. In 1980, Haynes et al. proposed a distinction between “typical” CS, as originally defined, and “atypical” CS (ACS), in which the ocular involvement does not affect the cornea but is responsible for chronic or recurrent conjunctivitis, scleritis, uveitis, optic disc edema, and retinal vasculitis [8]. It is more common for patients with ACS than for those with typical CS to have systemic manifestations [3]. A third disorder, which may have an overlapping pathogenesis and manifestations, is autoimmune inner ear disease (AIED), defined as primary when the pathology is restricted to the ear and secondary when it occurs in the context of a systemic autoimmune disease [9, 10]. All three syndromes (CS, ACS, and AIED) include neurological disorders, mostly as unexpected manifestations of acute vascular accidents and/or transient ischemic events.

In this study, we prospectively investigated the alterations in brain glucose metabolism that may have been induced by mild, long-standing cerebral vasculitis in patients presenting with the aforementioned syndromes but no prior neurological symptoms and normal conventional neuroimaging findings. Functional mapping of the cerebral glucose consumption was obtained by quantitative molecular imaging with 2-[18F]fluoro-2-deoxy-D-glucose (FDG) positron emission tomography (PET), combined with computed tomography (CT). detecting in vivo cerebral glucose utilization and changes in brain metabolism.

## 2. Patients and Methods

The study was conducted in its entirety at the University Hospital of Parma (Italy) with the participation of the following hospital units: Inflammatory and Infectious Ocular Diseases; Otorhinolaryngology; Nuclear Medicine; Medical Physics; and Neurology. The protocol was approved by the Institutional Ethics Committee, and informed consent was obtained from all patients according to the tenets of the Declaration of Helsinki. The patients included in the study were divided into three groups:

1. CS (i.e., typical disease, with interstitial keratitis)
2. ACS (autoimmune ocular inflammation other than interstitial keratitis)
3. AIED (primary and secondary)

The unmatched control group (CG) consisted of a cohort of individuals with a negative history of neuropsychiatric and neurological disorders who strictly required FDG-PET/CT for a pathology not involving the head and neck (mostly primary abdominal neoplasm) and who agreed to have the scan extended to the cerebral region (in which case the conventional written consent form was amended accordingly).

The enrollment procedure targeted patients diagnosed with unilateral or bilateral sensorineural hearing loss who had been referred to an ophthalmologist for the assessment of possible concomitant ocular inflammation. A complete medical history was obtained from each patient. Blood samples were collected and tested for autoimmunity inducers. CS or ACS was diagnosed by the patient's ophthalmologist and/or otolaryngologist based on the clinical features, general investigations to narrow the differential diagnosis, and results of serology for specific markers [11, 12]. AIED was finally diagnosed by an otolaryngologist after syphilis, noise and head trauma, drug toxicity, and hereditary hearing impairment were excluded. All patients also underwent brain magnetic resonance imaging (MRI) with gadolinium to exclude acoustic neurinoma, metastatic disease, lymphoma, and multiple sclerosis.

After a patient was assigned to one of the three study groups but before administration of any immunomodulatory treatment, a FDG-PET/CT scan was proposed and subsequently performed in all patients who provided specific written consent.

### 2.1. PET/CT Imaging and Analysis

18F-Fluorodeoxyglucose PET/CT was performed using the whole-body hybrid system “Discovery ST” (GE Healthcare, Chicago, USA) operating in three-dimensional detection mode. A head holder was used to restrict patient movement, which was nonetheless checked on a regular basis. The patients fasted for at least 6 h before radiopharmaceutical injection to ensure that their measured blood glucose level was <120 mg/dL. 18F-FDG (185–250 MBq) was administered intravenously in a quiet, dimly lit examination room. Brain PET/CT recording was started 30 min after tracer injection. During the 30-minute uptake period, the patients were left undisturbed in a darkened room and instructed to rest quietly without activity and with their eyes closed. Brain CT was recorded first to provide the attenuation correction map (120 kV; 150 mAs; 512 × 512 matrix; 3.75 mm slice thickness; scan type: helical pitch 1; number of images: 47; recon type: standard). It was immediately followed by a 3D-PET recording during a 10-minute period (field of view: 15.4 cm, recon type: iterative with 21 subsets and 2 iterations; 256 × 256 matrix; axial resolution: 4.8 mm at full-width half-maximum, FWHM). Patients were also scanned from the head to the mid thighs 45 minutes after tracer injection. The whole-body PET component was performed with a 3-minute acquisition per bed position with the scanner operating in the 3D mode. Normally, scans of 5–7 bed positions were obtained.

Quantitative analysis of brain PET was performed using the SPM5 software (Wellcome Trust Centre for Neuroimaging, London, UK, http://www.fil.ion.ucl.ac.uk/spm) implemented in Matlab R2014a [13]. The PET dataset was spatially normalized using the SPM5 PET template and smoothed with a Gaussian filter of 8 mm at FWHM. Statistical parametric mapping (SPM) was carried out to identify areas of significant hypometabolism in affected patients. Differences in the rate of cerebral glucose consumption (CMRglc) were assessed for a comprehensive group consisting of all of the affected patients (disease group, DG) and for disease-specific groups (CS, ACS, and AIED) comprising the patients with those diseases. These results were compared with those of the control group (CG) on a voxel-by-voxel basis using a two-sample *t*-test (SPM-*t*). Hypometabolic brain areas were investigated at a very high level of significance (*p* = 0.0001). Only clusters containing >100 voxels were considered to be significant. The results are displayed in the Talairach Atlas [14].

## 3. Results

During the study period (January 2012–September 2015), 44 Caucasian patients (25 males and 19 females, mean age 43 ± 14 years) were deemed eligible for the study and assigned to the three study groups as follows: 8 patients (4 males and 4 females, mean age 36 ± 15 years) were assigned to the CS group; 21 patients (13 males and 8 females, mean age 42 ± 13 years) were assigned to the ACS group; and 15 patients (8 males and 7 females, mean age 47 ± 16 years) were assigned to the AIED group. The demographic characteristics of the three study groups did not significantly differ (*p* > 0.05). The 16 patients (11 males and 5 females, mean age 63 ± 13 years) in the CG group were significantly older than the study patients (*p* < 0.01).

**Figure 1:**
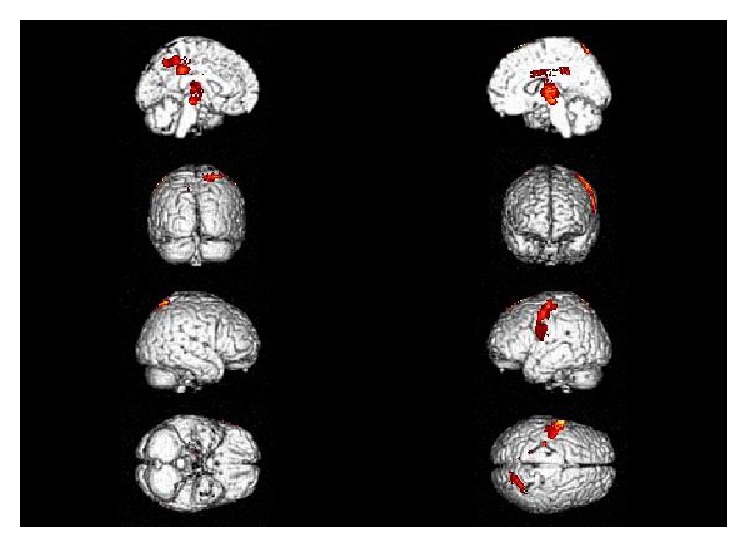
SPM hypometabolic maps comparing all the affected patients (DG) versus the control group (CG) overlaid on the structural MR template included in SPM5.

SPM-*t* maps of CMRglc generated from the PET images of the DG (i.e., all affected patients who underwent a PET examination) showed glucose hypometabolism in the following brain regions: cingulate cortex, precuneus, left precentral gyrus, right thalamus and lentiform nucleus, right substantia nigra, and superior parietal lobule (see Table 1 for the exact coordinate peaks and their localization). In Figure 1 SPM maps of CMRglc in the DG are compared to those in the CG overlaid on the structural MR template included in SPM5.

**Figure 2:**
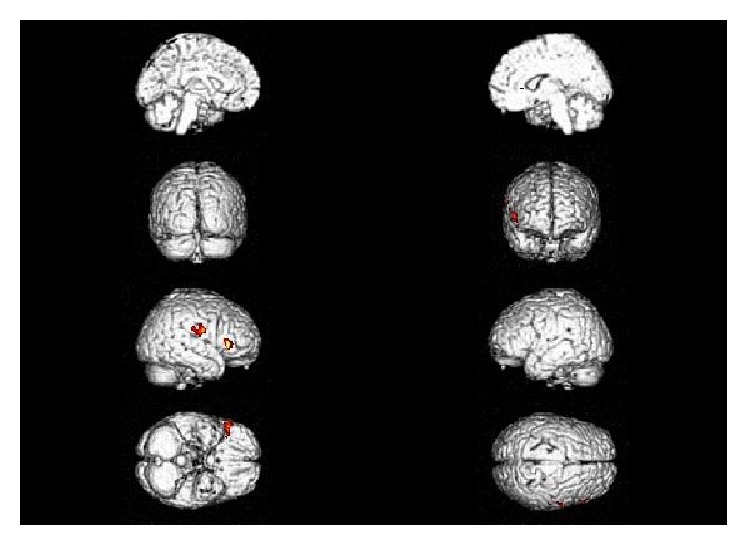
SPM hypometabolic maps comparing typical CS group and the control group.

The SPM-*t* maps of the CS patients showed glucose hypometabolism in the frontal lobe bilaterally, in the left precentral gyrus, and in the right parietal lobe (see Table 2 for the exact coordinate peaks and their localization and Figure 2 for the structural MR template).

**Figure 3:**
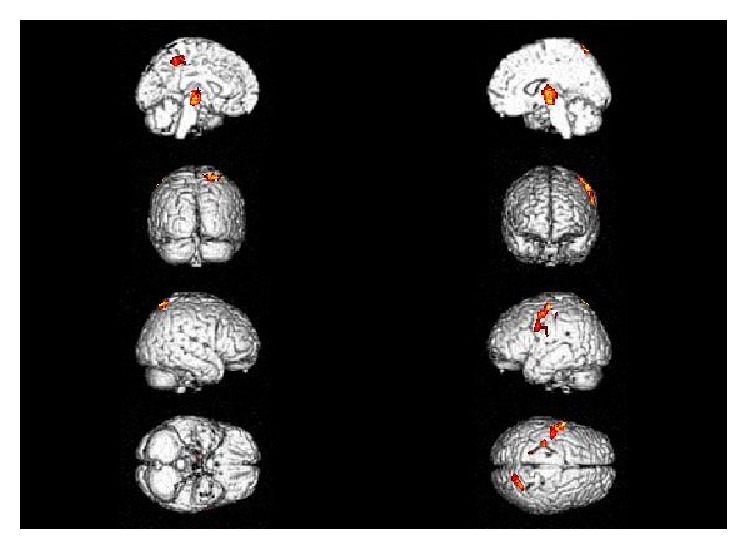
SPM hypometabolic maps comparing atypical CS group and the control group.

For the ACS patients, the SPM-*t* maps revealed a metabolic decrease involving the left frontal lobe, the parietal cortex bilaterally, the right thalamus and subthalamic nucleus, the left substantia nigra, and the left lentiform nucleus (see Table 3 and Figure 3 for details).

The SPM results for the AIED group are reported in Table 4. The PET/CT whole-body scan did not reveal any vascular uptake in the studied patients.

## 4. Discussion

Vasculitis in Cogan's syndrome (CS/ACS) affects variable-sized vessels. Severe neurological symptoms are reported in approximately 29% of patients and include headache, psychosis, coma, convulsion, neuropathy, and stroke. Cerebral vasculitis has been demonstrated in 12–15% of CS/ACS patients [15, 16]. Conventional imaging may show areas of ischemic change or infarction, meningoencephalitis, cerebral venous sinus thrombosis, and cranial neuropathy. MRI and CT may additionally show obliteration or narrowing of the vestibular labyrinth. On contrast-enhanced T1-weighted imaging, the membranous labyrinth may be enhanced [17]. In our patients' cohort, however, neurologic symptoms were absent or very mild, such that conventional neuroimaging does not show significant or specific alterations. FDG-PET/CT was recently used in CS patients to diagnose extracerebral vasculitis, by taking advantage of the overexpression of glucose transporters in activated inflammatory cells [18, 19]. The use of the whole-body scanning via FDG-PET may provide a sensitive metabolic imaging modality to visualize the early onset of inflammatory processes in large-vessel vasculitis (e.g., giant cell arteritis and Takayasu arteritis). Visual scoring of vascular [18F-]FDG-uptake compared to the liver FDG-accumulation was validated to represent the severity of inflammation, as recently confirmed by a meta-analysis [20–22].

In our cohort of patients with Cogan's syndrome the whole-body scan did not reveal any vascular uptake, excluding the presence of large-vessel inflammation. FDG-PET was so applied to measure CMRglc as a surrogate for neuronal activity. Several studies proposed an association between inflammatory activity and neurodegeneration, in both animal models and human subjects [23–25]. In the central nervous system, inflammatory processes activate microglial cells, which are responsible for releasing of proinflammatory cytokines including interleukin- (IL-) 1*β*, IL-6, and tumor necrosis factor-*α* [26, 27]. In turn, the proinflammatory cytokines aggravate and propagate neuroinflammation, thus leading to degeneration of healthy neurons, brain function impairment, decreased cerebral blood flow, and chronic cerebral hypoperfusion. This neuronal damage and these dysfunctions may cause glucose hypometabolism in different brain areas. Cerebral hypoperfusion and glucose hypometabolism are pathological mechanisms involved in both grey and white matter atrophy, cognitive decline, and Alzheimer's disease [28]. Abnormalities of brain glucose utilization were also studied in autoimmune vasculitis such as systemic lupus erythematosus. PET imaging was found to be a sensitive tool to detect manifest or subclinical central nervous system involvement, even earlier than MRI [29, 30].

No data are available on brain glucose metabolism in patients with CS. In this study, we prospectively investigated possible abnormalities in CMRglc first in the whole cohort of affected patients (DG) and then in patients assigned to each of the CS subforms (CS, ACS, and AIED). The results were compared with those obtained for the CG. SPM-*t* maps of DG showed regions of significant glucose hypometabolism in both hemispheres. These areas were the same as those that were altered in patients who presented with mild cognitive impairment progressed to Alzheimer's disease [28, 31].

A comparison of CS and CG identified six areas of significant hypometabolism, mostly localized in the right frontal and parietal lobes. The most extended hypometabolism was detected in ACS patients (11 regions); the regional distribution was similar to that seen in CS patients, but it was localized bilaterally and it involved more subcortical regions. The AIED group showed only few regions with glucose hypometabolism exclusively in the right hemisphere. The limitations of the study concern primarily (a) the limited number of patients, (b) the use of an age-unmatched control group (CG, older the study patients), and (c) the lack of an analytical neuropsychological assessment of the enrolled patients. CS is a very rare disease, and only with the assistance of the National Reference Center of our department could even this limited cohort of patients be enrolled. We tried to balance the lack of a matched CG and thus the risk of positive false results, by selecting a very high statistical threshold in the SPM analysis (*p* = 0.0001).

## 5. Conclusion

This study revealed areas of significantly altered brain glucose consumption in a cohort of patients with a first diagnosis of CS (any subform) and no evidence of neurologic symptoms or conventional neuroimaging alterations. Our preliminary results suggest that a PET/CT examination may represent a very useful tool for the global assessment of CS patients, either by excluding the inflammation of the large-sized vessels or by characterizing brain glucose metabolism. This could also help in adjusting immunosuppressive treatment. We recommend assessing the cognitive function of these patients by performing specific neuropsychological tests at the baseline and during the follow-up.

## Figures and Tables

**Table 1:** Coordinates of Talairach's Atlas (*x*/*y*/*z* axis) resulting from hypometabolic contrast SPM-*t* analysis of all the affected subjects (DG) versus control group.

*x*	*y*	*z*	Area	Left/right
−4.0	−31.0	37.0	Limbic lobe cingulate gyrus	L
−16.0	−42.0	50.0	Parietal lobe precuneus	L
−53.0	−1.0	48.0	Frontal lobe precentral gyrus	L
22.0	−13.0	14.0	Thalamus	R
28.0	−25.0	3.0	Lentiform nucleus	R
14.0	−18.0	−8.0	Brainstem midbrain substantia nigra	R
34.0	−53.0	67.0	Parietal lobe superior parietal lobule	R

**Table 2:** Coordinates of Talairach's Atlas (*x*/*y*/*z* axis) resulting from hypometabolic contrast SPM-*t* analysis of typical Cogan's syndrome group versus control group.

*x*	*y*	*z*	Area	Left/right
51.0	27.0	4.0	Frontal lobe inferior frontal Gyrus	R
−34.0	−14.0	28.0	Frontal lobe precentral gyrus	L
51.0	−9.0	21.0	Parietal lobe postcentral gyrus	R
59.0	−18.0	25.0	Parietal lobe postcentral gyrus	R
34.0	−12.0	32.0	Frontal lobe precentral gyrus	R
26.0	−23.0	44.0	Frontal lobe precentral gyrus	R

**Table 3:** Coordinates of Talairach's Atlas (*x*/*y*/*z* axis) resulting from hypometabolic contrast SPM-*t* analysis of atypical Cogan's syndrome group versus control group.

*x*	*y*	*z*	Area	Left/right
−57.0	2.0	31.0	Frontal lobe precentral gyrus	L
−51.0	−1.0	48.0	Frontal lobe precentral gyrus	L
−44.0	−11.0	58.0	Frontal lobe precentral gyrus	L
22.0	−61.0	66.0	Parietal lobe superior parietal lobule	R
−16.0	−42.0	32.0	Parietal lobe precuneus	L
−32.0	−23.0	47.0	Parietal lobe postcentral gyrus	L
−18.0	−32.0	51.0	Frontal lobe paracentral lobule	L
18.0	−13.0	8.0	Thalamus	R
14.0	−16.0	−6.0	Brainstem midbrain, gray matter subthalamic nucleus	R
−20.0	−13.0	3.0	Lentiform nucleus	L
−10.0	−20.0	−6.0	Brainstem midbrain substantia nigra	L

**Table 4:** Coordinates of Talairach's Atlas (*x*/*y*/*z* axis) resulting from hypometabolic contrast SPM-*t* analysis of autoimmune inner ear disease group versus control group.

*x*	*y*	*z*	Area	Left/right
53.0	33.0	0.0	Frontal lobe inferior frontal gyrus	R
51.0	20.0	12.0	Frontal lobe inferior frontal gyrus	R
